# Automatic analysis of cochlear response using electrocochleography signals during cochlear implant surgery

**DOI:** 10.1371/journal.pone.0269187

**Published:** 2022-07-14

**Authors:** Sudanthi Wijewickrema, Christofer Bester, Jean-Marc Gerard, Aaron Collins, Stephen O’Leary

**Affiliations:** 1 Department of Surgery (Otolaryngology), University of Melbourne, Melbourne, Australia; 2 Royal Victorian Eye and Ear Hospital, Melbourne, Australia; Hannover Medical School: Medizinische Hochschule Hannover, GERMANY

## Abstract

Cochlear implants (CIs) provide an opportunity for the hearing impaired to perceive sound through electrical stimulation of the hearing (cochlear) nerve. However, there is a high risk of losing a patient’s natural hearing during CI surgery, which has been shown to reduce speech perception in noisy environments as well as music appreciation. This is a major barrier to the adoption of CIs by the hearing impaired. Electrocochleography (ECochG) has been used to detect intra-operative trauma that may lead to loss of natural hearing. There is early evidence that ECochG can enable early intervention to save natural hearing of the patient. However, detection of trauma by observing changes in the ECochG response is typically carried out by a human expert. Here, we discuss a method of automating the analysis of cochlear responses during CI surgery. We establish, using historical patient data, that the proposed method is highly accurate (∼94% and ∼95% for sensitivity and specificity respectively) when compared to a human expert. The automation of real-time cochlear response analysis is expected to improve the scalability of ECochG and improve patient safety.

## Introduction

### Overview

Hearing loss was the fourth most prevalent disease and the third leading cause of ‘Years Lost with Disability’ in 2016 according to the Global Burden of Disease report [1]. Its treatment can prevent downstream effects such as dementia [2] and socioeconomic disadvantages [3]. Disabling hearing loss affected 6.1% of the world’s population in 2017 [4]. A cochlear implant (CI) is a cost-effective solution [5] because it restores a recipient’s economic and social independence. But as yet, as few as 5% of adults who may benefit from this technology receive a CI [6]. An important reason for this is that many CI candidates fear losing their residual, natural hearing [7–9] as occurs after 50-70% of CI surgeries. If this natural hearing could be reliably preserved during CI surgery, more patients would benefit from the advantages a CI can offer.

Cochlear implantation has until recently been a ‘blind procedure’, where the surgeon was not able to monitor the condition of the inner ear during implantation. Any damage caused during the surgery could only be observed 2-3 weeks after the procedure using conventional hearing tests. However, intra-operative electrocochleography (ECochG) has made it possible to monitor the response of the cochlea to sound during surgery [10]. Previous studies have shown that changes in particular ECochG components can be used to predict the preservation of post-operative residual hearing [11, 12]. The process of trauma detection has so far necessitated the presence of a human expert. This requirement for a human expert’s presence during cochlear implantation is a significant barrier to the widespread use of intra-operative ECochG monitoring in clinical practice.

The real-time decisions made by a human expert have been demonstrated to predict hearing preservation in observational studies [13], as well as improve rates of hearing preservation when used to initiate intervention during implantation [14]. These results provide us with a validated approach to interventional cochlear implant guidance using electrocochleography. Here, we aim to automate this validated approach by analysing cochlear responses to achieve parity with the decisions made by a human expert, using multiple metrics derived from the ECochG response. We validate this method using historical patient data compared to the performance of a human expert.

### Real-time electrocochleography

In real-time ECochG, an acoustic stimulus is delivered to the ear as the CI electrode is being inserted into the cochlea. The acoustic stimulus is presented with alternating polarity that reduces (rarefact) or increases (condense) air pressure at stimulus onset. Modern real-time ECochG systems typically use the electrode itself as a recording device, rather than an extra-cochlear electrode, as this improves the signal to noise ratio [12]. The electrical potentials recorded are composed of contributions from both sensory (hair) cells and the auditory neurons.

**Fig 1:**
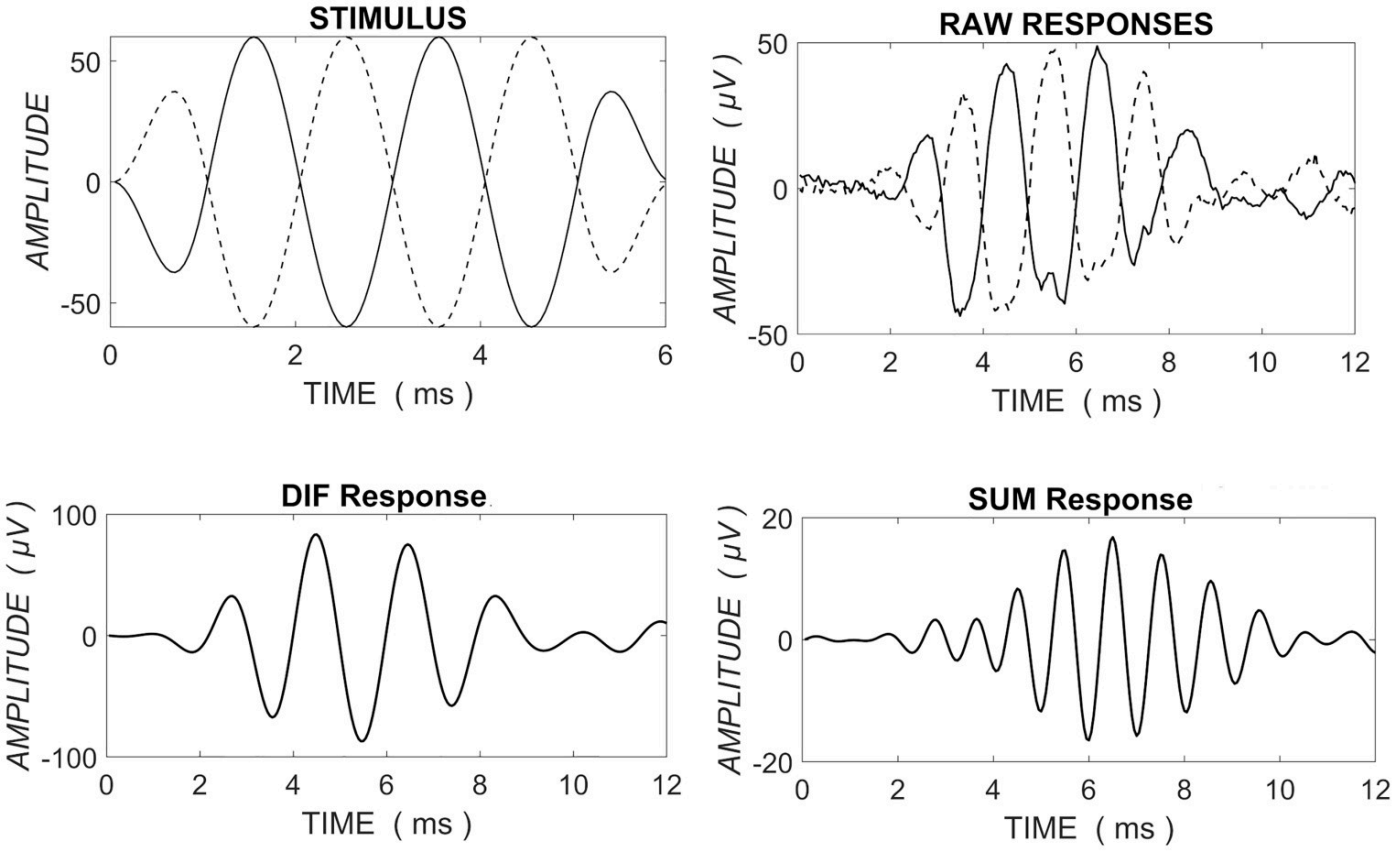
Top panels: left shows the stimulus of alternating polarity and right shows the raw responses recorded for both condensation and rarefaction stimuli. Bottom panels: DIF response calculated by subtracting rarefaction and condensation responses is on the left while the SUM calculated by the addition of the responses is on the right.

The response of the sensory (hair) cells is estimated by taking the difference of the alternating polarity responses (DIF). This is termed the ‘Cochlear Microphonic’ (CM) [15, 16]. The CM is *primarily* generated by receptor currents in outer hair cells (OHCs) [17, 18]. The amplitude (and phase) of the CM is measured by taking the Fast Fourier Transform (FFT) of the DIF response at the stimulus frequency, as the CM follows the stimulus. The neural response is estimated by taking the sum of the alternating polarity responses (SUM), which cancels frequency-following responses and leaves an estimation of the phase-locked ‘Auditory Nerve Neurophonic’ (ANN). As this phase-locking occurs preferentially as inner hair cells depolarize [19], it results in an asymmetric response which produces a large component of distortion at twice the stimulus frequency. The amplitude (and phase) of the ANN is therefore measured at the 2nd harmonic of the SUM [20]. Fig 1 shows an example of the signals measured using ECochG. Note that this approach to deriving the CM and ANN will not exclusively derive outer hair cell and neural contributions, and should be considered an estimation.

### Manual detection of trauma

**Fig 2:**
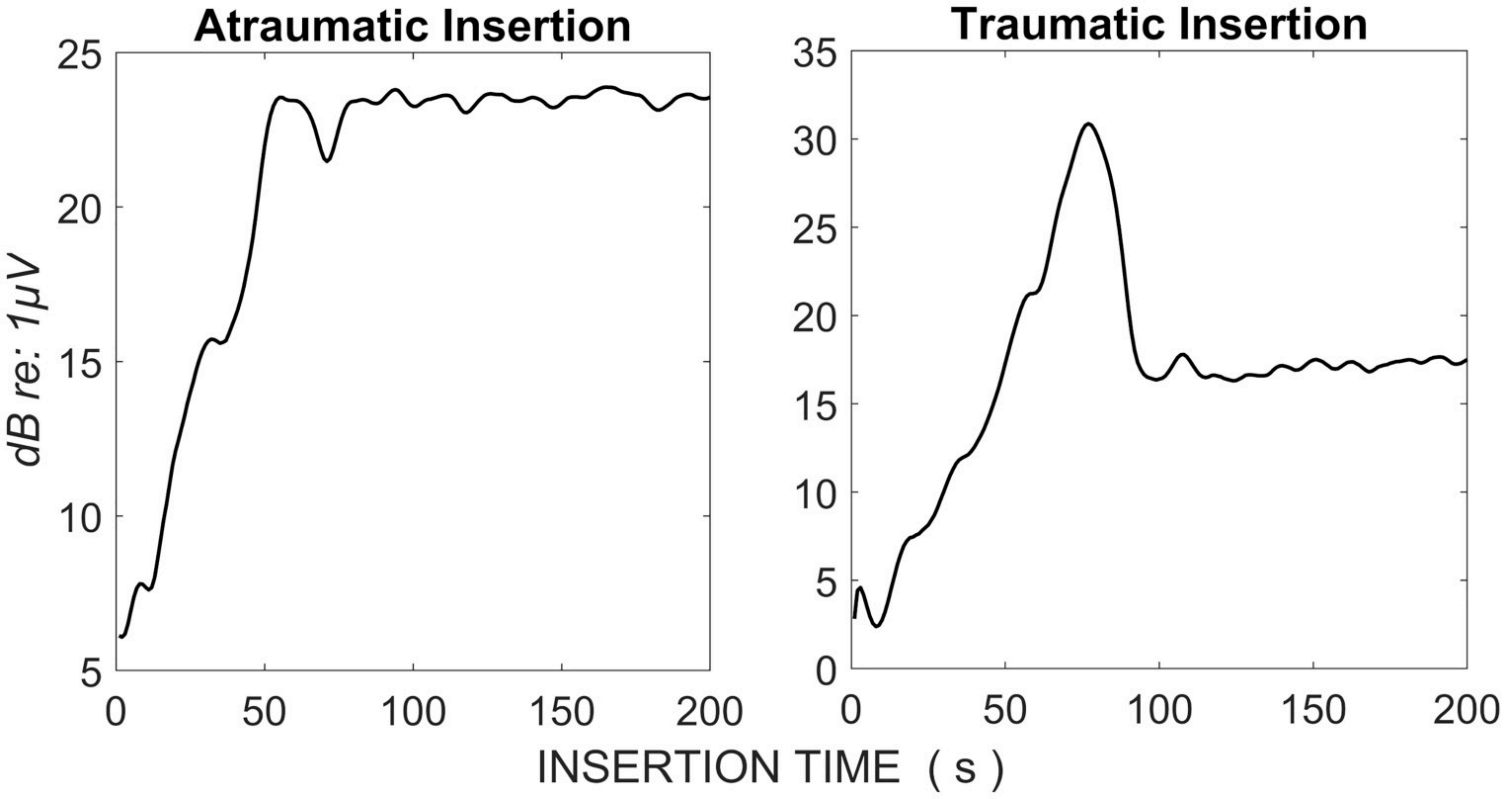
Changes in the cochlear microphonic for two patients with presumed electrophysiological evidence of atraumatic (left) and traumatic (right) CI insertions.

Intra-operative detection of trauma using ECochG has so far focused on amplitude changes of the CM, as the most readily detectable ECochG signal [11, 12]. For example, [11] demonstrated that a 30% drop in CM amplitude predicts poorer preservation of natural hearing [11]. Fig 2 shows the differences typically observed in the CM amplitudes for patients we define as having electrophysiological evidence of traumatic or atraumatic CI insertions. Note the sharp drop in the CM amplitude that occurred in the traumatic insertion that was indicative of trauma.

## Materials and methods

### Database

In order to develop and validate our automatic trauma detection method, we used the intra-operative ECochG recordings of 77 patients that underwent CI surgery. The 77 patients were drawn from 95 patients who had ECochG recorded in our department over 2017 and 2018. 18 patients were excluded due to low ECochG response amplitudes (<1*μV*). A 1 *μV* threshold for CM amplitude, specifically in the bandpass filtered DIF signal around the stimulus frequency (15th order digital bandpass filter around the stimulus frequency (0.9*F to 1.1*F)), was used as this is the minimum amplitude required to exceed the noise floor of our ECochG system by 3 standard deviations. In our experience, once exceeded, this allows sufficient signal to noise ratio for us to detect a 30 percent drop reliably. Written consent was obtained from each patient and data was anonymised to remove all identifying information. Ethics approval for the collection of this data was obtained by the Human Ethics Committee of the Royal Victorian Eye and Ear Hospital (HREC #14/1171H/19).

Our approach to ECochG has been described in detail previously [11]. Briefly, adult subjects with residual low-frequency hearing (hearing threshold ≤ 80dBHL at 0.5kHz) receiving Cochlear Limited’s Nucleus CI422 or 522 implants were enrolled in this study. ECochG was recorded using the Cochlear Response Telemetry system. The acoustic stimuli was a tone pip with a frequency of 0.5kHz and a duration of 12ms with 1ms linear on- and offset ramps, at a presentation rate of 14 per second. The intensity of the acoustic stimuli was 100-110 dB HL. The ECochG response was recorded from the electrode at the tip of the array, at recording windows of 12ms in duration at 20 kHz. Each waveform was averaged from 100 stimulus presentations. Once recorded, the amplitude of the DIF response was calculated by taking the magnitude of the DIF response at the stimulus frequency, here 0.5kHz. The amplitude was calculated by (FFT), by first zero-padding to 1000 samples for 20Hz bin size, and then taking the bin at the stimulus frequency.

An expert in ECochG labelled the data at two levels. First, each recording was labelled as a ‘drop’ or ‘no drop’ depending on whether any drops in the ECochG signal were observed or not. Second, in the patients whose recording had drops in the ECochG signal, the duration of each drop (from the time a human expert would start detecting it in real-time to the end of the drop) was identified. Each time point in each identified duration was then labelled as a ‘drop’ instance and all other time points were labelled as ‘no drop’ instances.

As drops, by definition, have to be on falling edges of the ECochG signal, all time points where the CM signal was rising or constant were removed from the data, in order to simplify the classification problem. The remaining patient data was randomly separated into 5 subsets ensuring each subset included at least one patient with a drop. These subsets were then used in a 5-fold cross validation for the training and testing of our trauma detection method.

### Experimental setup

All methods were implemented in MATLAB R2020a. Classifiers included in the Statistics and Machine Learning Toolbox were used in the experiments. All experiments were run on a computer with a 2.11GHz Intel Core i5 processor and 8GB of RAM. The operating system was 64-bit Windows 10.

### Features

We used features including those used by human experts in detecting drops and/or been shown in previous work to inform the presence of a drop in the ECochG signal. These features were derived from the CM at the fundamental frequency, 500Hz, and the corresponding ANN at 1KHz. The features considered were: the amplitude and phase of the CM and ANN signals, ratio of the CM and ANN amplitudes, the fraction of the CM amplitude with respect to the immediate previous peak in the signal, time from the immediate previous peak, and coefficient of variation (standard deviation / mean) of the CM amplitude in a window including the current instance and 4 immediately prior to that.

In order to determine the 2 peak-related metrics, we developed a real-time algorithm that detected peaks and troughs in an ECochG signal. First, we identified all local peaks and troughs. If at time *t*, the amplitude of the CM signal satisfies the conditions ‖*CM*‖_*t*−2_ < ‖*CM*‖_*t*−1_ and ‖*CM*‖_*t*−1_ ≥ ‖*CM*‖_*t*_, ‖*CM*‖_*t*−1_ is a peak. Similarly, ‖*CM*‖_*t*−1_ is a trough if ‖*CM*‖_*t*−2_ ≥ ‖*CM*‖_*t*−1_ and ‖*CM*‖_*t*−1_ < ‖*CM*‖_*t*_. Each peak thus detected and the trough that occurred immediately before that were paired together. Second, if a peak was detected, we identified if it was an active peak or a local fluctuation using the following conditions. The current peak was considered to be an active peak if:

- There were no active peaks detected so far, or
- The signal at the current peak is larger than the last active peak, or
- The signal at the current peak is larger than *s*_*a*_ times the last active peak, and the amplitude difference between the current peak and trough is larger than *s*_*b*_ times the current peak, where *s*_*a*_ and *s*_*b*_ are scalars defined as 0.5 and 0.1 respectively through trial and error.

**Fig 3:**
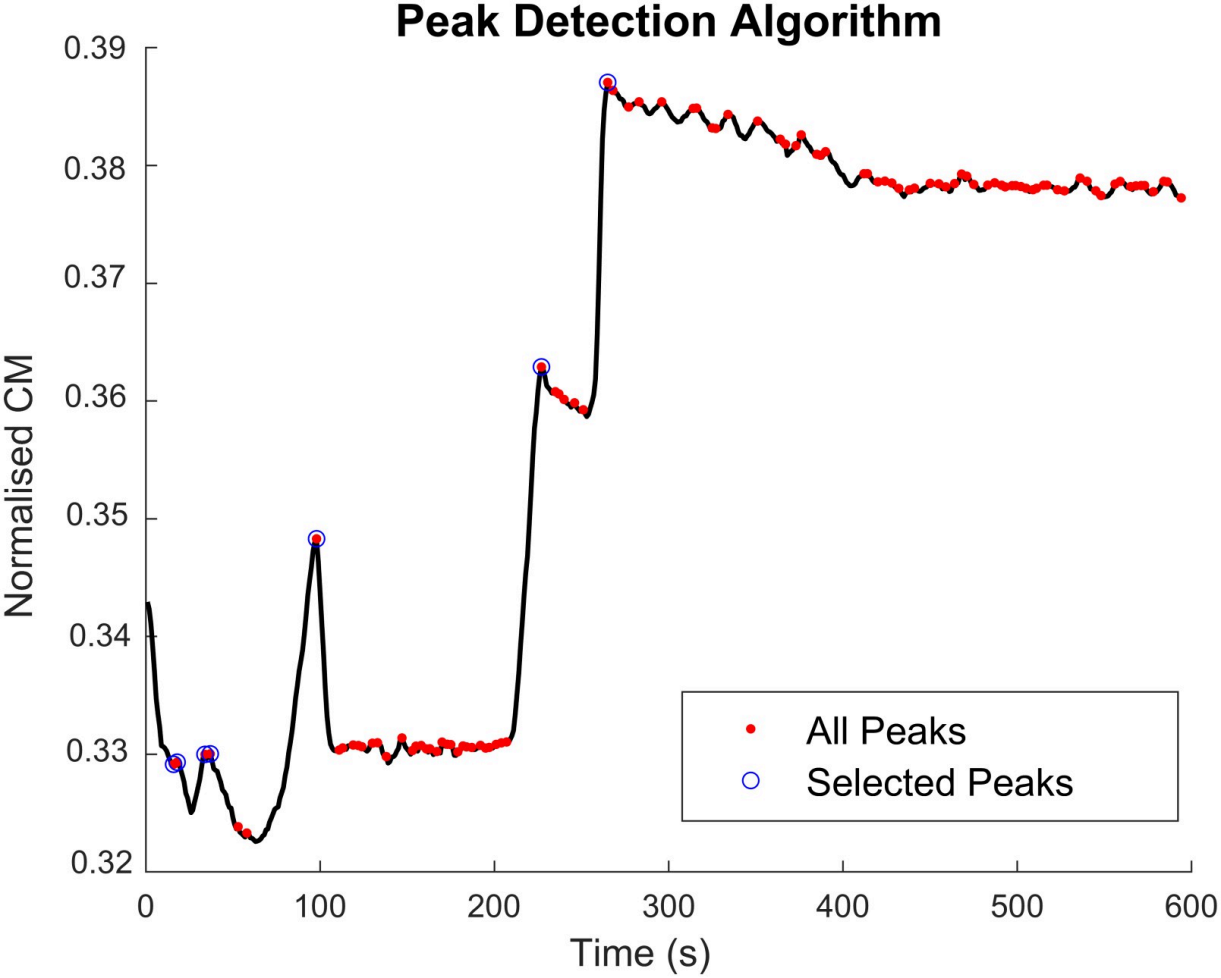
Results of the real-time peak detection algorithm. All peaks of the CM signal are shown as red dots. The peaks detected by the algorithm as those an expert would pick in determining drops are shown in blue circles.

Fig 3 shows the results of the peak detection algorithm on the CM signal of a patient in our dataset. The set of features *ft*_1..8_ used to detect drops in the ECochG signal at time *t* were thus defined as shown in Eq 1. *ϕ* denotes the phase of a CM or ANN signal, and *pk* is the current active peak.

$$\begin{array}{c} \begin{array}{cc} ft_{1} & ={∥CM∥}_{t}\\ ft_{2} & =\phi {\left(CM\right)}_{t}\\ ft_{3} & =∥ANN∥\\ ft_{4} & =\phi {\left(ANN\right)}_{t}\\ ft_{5} & =\frac{{∥CM∥}_{t}}{{∥ANN∥}_{t}}\\ ft_{6} & =\frac{{∥CM∥}_{t}}{{∥CM∥}_{pk}}\\ ft_{7} & =t_{pk}-t\\ ft_{8} & =\frac{mean(∥CM{∥}_{t-4\to t})}{std(∥CM{∥}_{t-4\to t})} \end{array} \end{array}$$ (1)

In addition to these primary features (derived from the CM at 500Hz and ANN at 1KHz), we determined the same for their harmonics at different frequencies (CM at 1KHz, 1.5KHz, and 2KHz and ANN at 2KHz, 3KHz, and 4KHz). We also considered a window of 3 time points (starting at 2 points prior to the current time point) and used the features calculated at these points in the feature set in order to test if previous readings would contribute to the detection of trauma. As such, we considered 96 features in total for each time point.

### Data normalisation

The potentials recorded with ECochG vary substantially between patients and are often normalised [21, 22]. We used the values of the initial ECochG readings as a baseline to normalise amplitude data. To this end, we calculated the mean of the CM/ANN amplitude of the first 5 data points and subtracted this value from every subsequent amplitude reading. We used the *sin* value of the phase angle for CM and ANN to ensure continuity of data. For each fold of the 5-fold cross validation, we then normalised each feature (in both training and test sets) to be in the range [01] using the minimum and maximum values of the corresponding feature in the training data.

### Drop classification

We trained and tested several classification methods using the above features in order to detect if a drop in the ECochG signal occurred at a given time point. The classifiers thus trained were: tree ensembles (TE) [23], discriminant analysis (DA) [24], naive Bayes (NB) [25], support vector machines (SVM) [26], k-nearest neighbours (KNN) [27], and neural networks (NN) [28].

We used different variants of these classifiers. To create tree ensembles, we used adaptive boosting (AdaBoost) [29], random under sampling boosting (RUS) [30], and bootstrap aggregation bagging (Bag) [31] algorithms. For discriminant analysis, we used linear and quadratic functions. In Naive Bayes, we used 2 different ways of calculating the probability density function: using a normal distribution (Gaussian) and kernel density estimation (Kernel). When using support vector machines, we employed linear, quadratic, cubic, and Gaussian kernel functions. We considered 3 variations of the Gaussian kernel with different *σ* values (Fine: 1.1, Medium: 4.5, and Coarse: 18). In k-nearest neighbour classification, we used different numbers of neighbours and distance functions (Fine, Medium, and Coarse: Euclidean distance with 1, 10, and 100 neighbours respectively, Cosine and Cubic: 10 neighbours with Cosine and Minkowski distances respectively, and Weighted: 10 neighbours with weighted Euclidean distance). We defined our artificial neural network as a feedforward (FF) network with one hidden layer of 10 neurons.

### Post processing

To identify obviously misclassified observations, we implemented a post-processing algorithm. This algorithm compared the current data (and the corresponding classification) to that seen previously in the same patient. The benefits of this strategy are twofold: 1) it can be implemented in real-time and 2) the variability of the between-patient CM amplitudes due to variations in natural hearing (which is necessarily present in the classification stage) can be avoided. The following conditions were used to correct misclassification of points.

- If the previous point on a falling edge (of the ∥*CM*∥) was classified as a ‘drop’, the current point is also a ‘drop’.
- If a point on a falling edge is classified as a ‘drop’ but the standard deviation of the ∥*CM*∥ in a window including that point and 4 previous points is less than *s*_*c*_ times the minimum ∥*CM*∥ in that window, it is a ‘no drop’.

**Fig 4:**
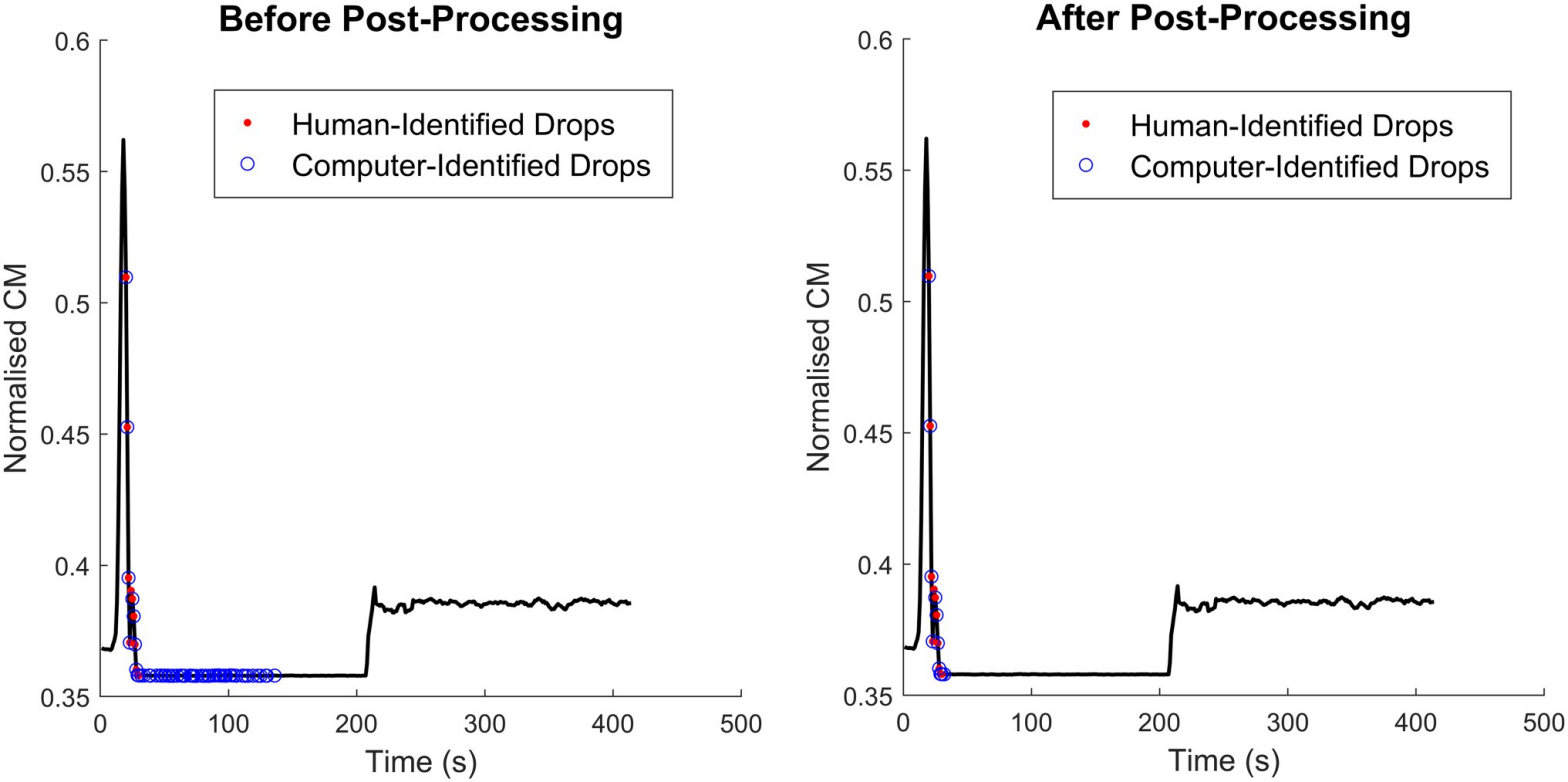
Results of the post-processing algorithm. The left panel shows the classification results prior to post-processing. The right panel shows how the post-processing algorithm has removed some of the misclassifications. Drop points detected by the human expert are given as red dots while those detected by the automated algorithm are given in blue circles.

The scalar *s*_*c*_ was determined through trial and error to be 0.01. Fig 4 shows the results of this algorithm for a patient in the test set of one of the folds in our cross-validation dataset.

### Performance metrics

In order to evaluate the performance of the classification methods, we used the commonly used metrics of sensitivity (the ability to correctly detect ‘drops’) and specificity (the ability to correctly detect ‘no drops’) [32]. We also calculated the overall accuracy of the classification. Eq 2 shows how these metrics were calculated.

$$\begin{array}{c} \begin{array}{cc} \text{Sensitivity} & =\frac{\#\text{True}\;‘\text{Drops}’}{\#\text{True}\;‘\text{Drops}’+\#\text{False}\;‘\text{No}\;\text{Drops}’}\\ \text{Specificity} & =\frac{\#\text{True}\;‘\text{No}\;\text{Drops}’}{\#\text{True}\;‘\text{No}\;\text{Drops}’+\#\text{False}\;‘\text{Drops}’}\\ \text{Accuracy} & =\frac{\#\text{True}\;‘\text{Drops}’+\#\text{True}\;‘\text{No}\;\text{Drops}’}{\#\text{All}\;\text{Falling}\;\text{Edge}\;\text{Points}} \end{array} \end{array}$$ (2)

**Fig 5:**
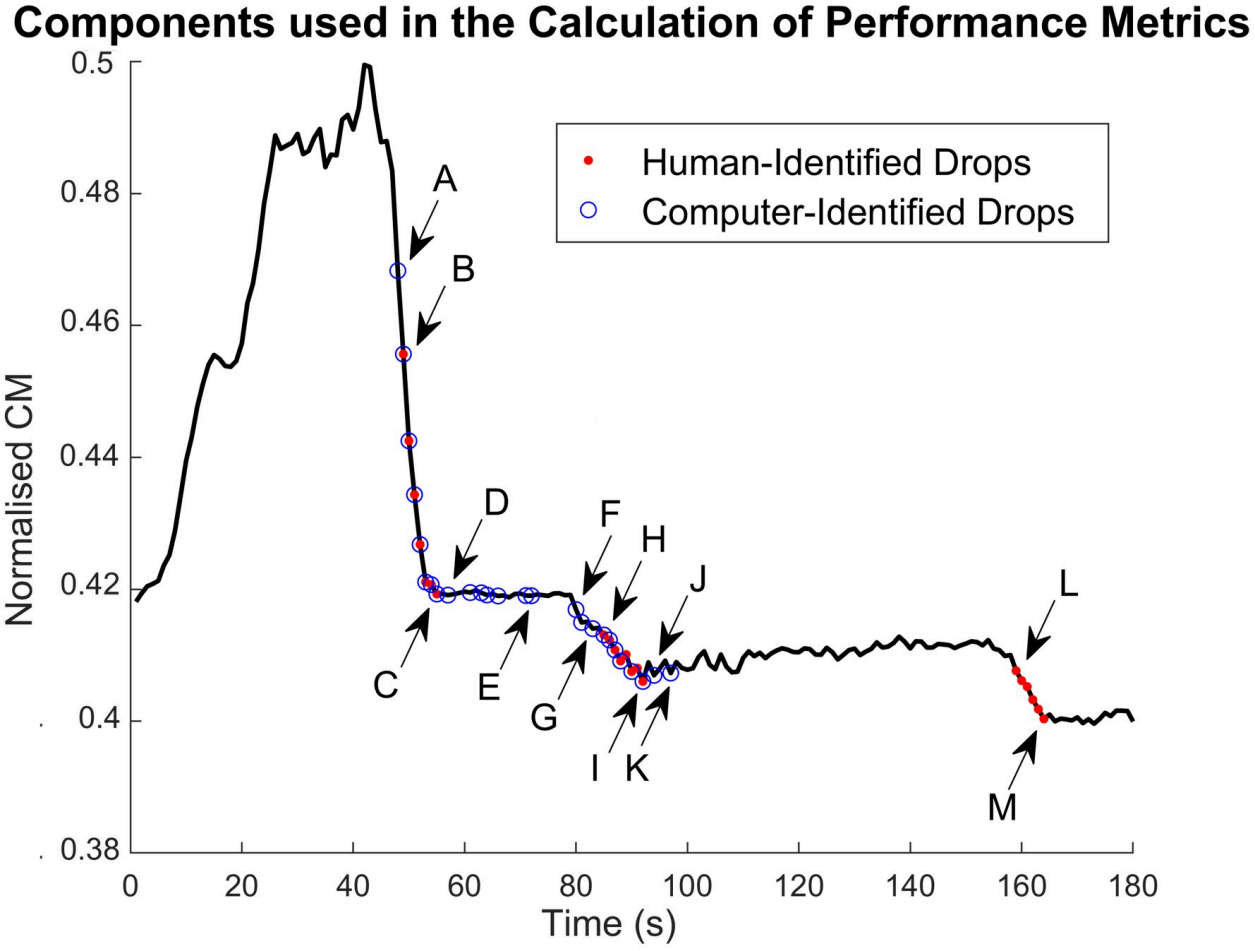
Components used in the calculation of performance metrics. B-C and H-I are True ‘Drops’. L-M are False ‘No Drops’. D-E and J-K are False ‘Drops’. A and F-G are drops identified before the human expert and therefore are considered to be True ‘Drops’. All other instances are True ‘No Drops’.

Fig 5 shows an example of the different components used in the calculation of performance metrics. Detecting a drop on a falling edge before a human would is not only acceptable but also beneficial. However, these points are counted as false ‘No Drops’ when comparing human and computer generated results. To overcome this issue, we reclassified such points as true ‘Drops’.

## Results

### Feature correlation

**Fig 6:**
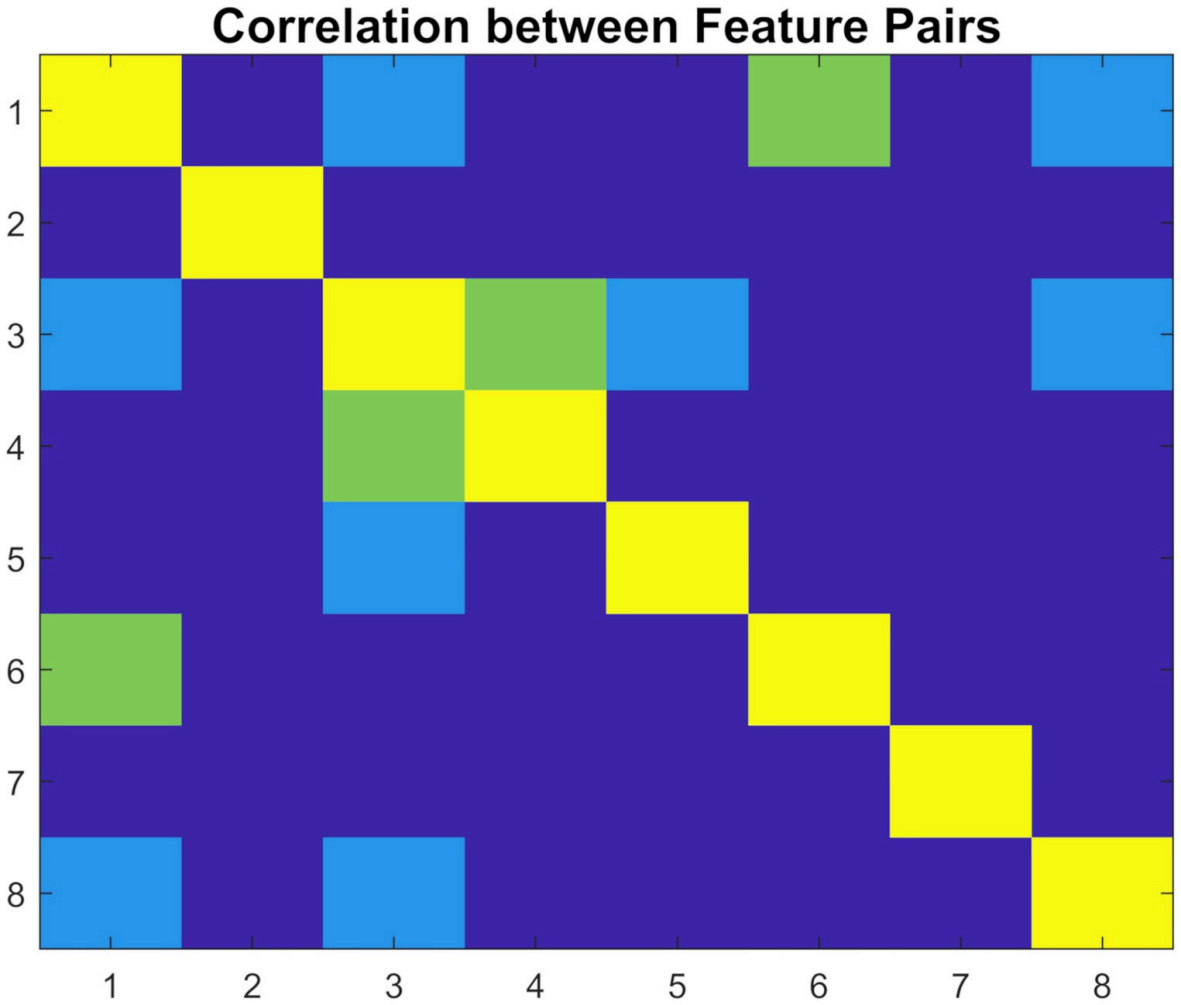
Correlation of feature pairs as measured using Pearson’s correlation coefficient. Yellow indicates perfect correlation (on the diagonal between the same features). Green, light blue, and dark blue show the pairs with moderate, low, and negligible correlations respectively.

We calculated the pair-wise linear correlation between features to determine if there were any dependencies between them. We observed that the Pearson’s correlation coefficient (*r*) between features was negligible (|*r*| < 0.25) for 22 feature pairs, weak (0.25 ≤ |*r*| < 0.5) for 4 feature pairs, and moderate (0.5 ≤ |*r*| < 0.75) for 2 feature pairs. Fig 6 shows the correlations between feature pairs. Since there were no strong correlations (|*r*| > 0.75) between features, we used all 8 features to train our classifiers.

### Selection of a classification method

We first trained the above mentioned classifiers on the primary features at time *t* (not considering features derived from the harmonics or those from previous time points) in order to select the best classifier for our purpose. As the dataset was unbalanced, we biased the training towards the detection of drops by increasing the penalty for misclassifying drops. As an initial value for this penalty term, we used the ratio between the number of ‘no drop’ and ‘drop’ instances (∼22). Test results of the classification (the average of the 5-fold cross validation) are shown in Table 1. Four classifiers (AdaBoost tree ensemble, support vector machines with quadratic and Gaussian (*σ* = 18) kernels, and k-nearest neighbours with Eucledean distance and 100 neighbours) showed high performance levels (>0.9 for all 3 performance metrics). From these 4 classifiers, we selected the one with the highest accuracy (AdaBoost tree ensemble) to be used in the next stages of the process.

**Table 1:** Performance of different classifiers in detecting drops in ECochG.

Model		Sensitivity	Specificity	Accuracy
TE	ADABoost	**0.9147**	**0.9520**	**0.9504**
	RUS	0.8616	0.9606	0.9563
	Bag	0.6900	0.9806	0.9686
DA	Linear	0.7670	0.9479	0.9398
	Quadratic	0.7736	0.9558	0.9480
NB	Kernel	0.8886	0.9411	0.9387
	Gaussian	0.7945	0.9485	0.9421
SVM	Linear	0.8968	0.9022	0.9020
	Quadratic	**0.9197**	**0.9432**	**0.9421**
	Cubic	0.7666	0.9684	0.9599
	Fine Gaussian	0.6577	0.9788	0.9658
	Medium Gaussian	0.9026	0.9445	0.9427
	Coarse Gaussian	**0.9420**	**0.9090**	**0.9105**
KNN	Fine	0.6139	0.9814	0.9666
	Medium	0.8475	0.9524	0.9478
	Coarse	**0.9122**	**0.9229**	**0.9224**
	Cosine	0.8299	0.9598	0.9542
	Cubic	0.8430	0.9530	0.9481
	Weighted	0.8475	0.9529	0.9483
NN	FF	0.7755	0.8529	0.8491

The results are the average of the 5-fold cross validation. Classifiers with all 3 metrics above 0.9 are shown in bold.

### Refinement of classifier

We observed that when the classifier was trained using the full 94 features (including features from harmonics as well as the full time window), it did not significantly improve classification results (0.8971, 0.9563, and 0.9537 for sensitivity, specificity, and accuracy respectively). Therefore, only the primary features were used in the classification.

**Fig 7:**
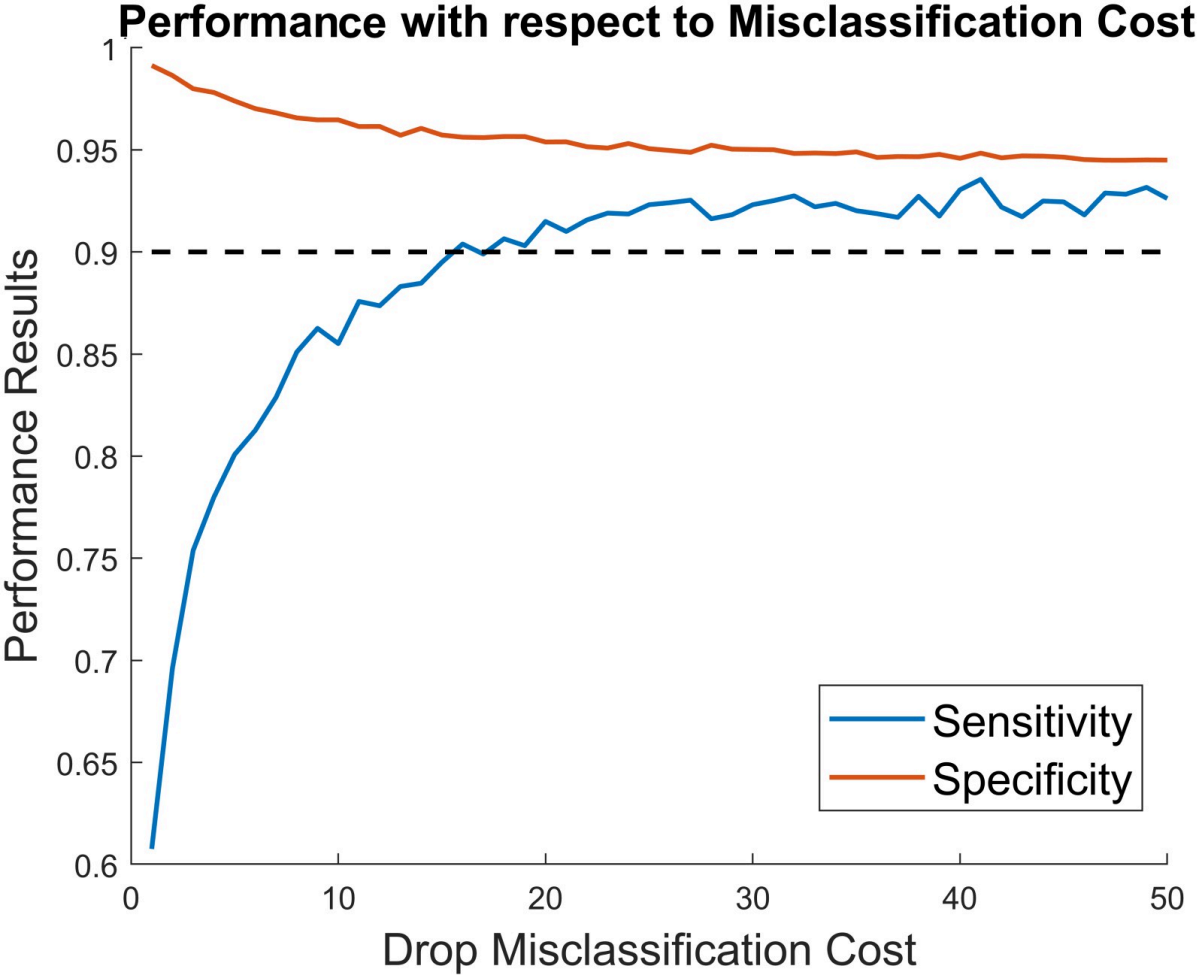
Effect of drop misclassification cost on performance results. Changes in sensitivity and specificity when the drop misclassification cost is increased is shown in blue and red respectively. The black dashed line indicates the cut-off level of 0.9 which was deemed acceptable for both metrics.

We determined the ideal misclassification cost (with respect to sensitivity and specificity) using an iterative search. Fig 7 shows the results of this search. We selected 41 as the best misclassification cost as it maximised both sensitivity and specificity. The final results were 0.9356, 0.9484, and 0.9478 for sensitivity, specificity, and accuracy respectively. The average prediction time per instance (for peak-detection, classification, and post-processing) was ∼0.75 ms.

### Feature importance

**Fig 8:**
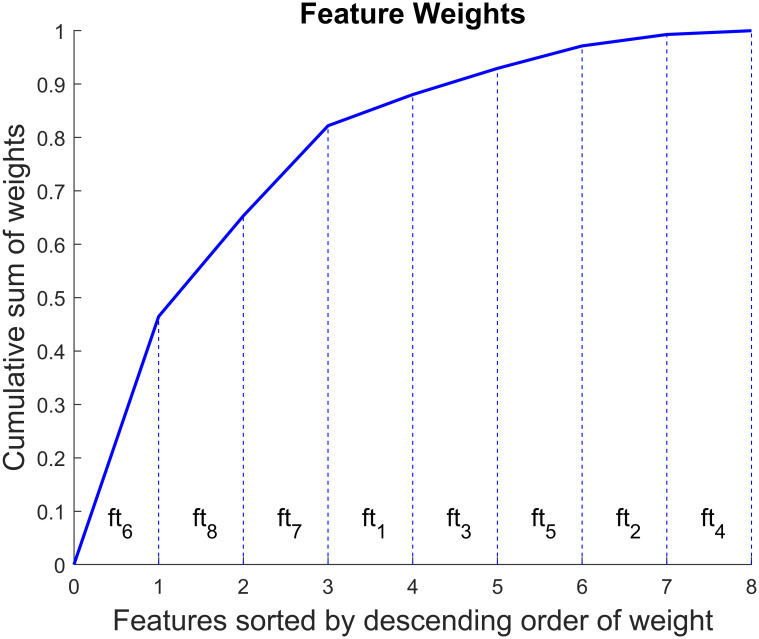
Feature importance in drop classification. The feature numbers refer to those defined in Eq 1. The horizontal axis shows the features sorted in descending order of importance (or weight). The vertical axis shows the cumulative sum of the feature weights.

We obtained the importance of the different features for the selected classifier. To this end, we calculated the average weights across the 5 folds for each feature. The feature that explained about 47% of the classification results was the ratio of ‖*CM*‖ to the previous peak. The coefficient of variation of ‖*CM*‖ and time from the previous peak contributed similarly (∼19% and ∼17% respectively). ‖*CM*‖ ‖*ANN*‖, and ‖*CM*‖:‖*ANN*‖ attributed for about 6%, 5%, and 4% respectively. *ϕ*(*CM*) and *ϕ*(*ANN*) accounted for only ∼2% and ∼1% of the results respectively. Fig 8 shows the cumulative importance of the features.

## Discussion

Intra-operative monitoring of inner-ear health during cochlear implantation is a rapidly advancing method for predicting the preservation of residual hearing [11, 12]. Thus far, these methods have been largely observational, and when used to modify surgical approach, have been done as ad-hoc, surgeon-driven processes without consistency of approach or a dedicated tool [33]. The common end-goal of clinical research into intra-operative ECochG is to automatically and rapidly provide feedback in the operating theatre, removing the need for an expert observer. To advance this approach, we introduced here a framework for automatically detecting insertion trauma using a machine learning algorithm.

Prior attempts at improving the sensitivity and specificity of trauma detection with intra-operative ECochG have used human-picked features (for example, Weder et al. [34]), with a maximum sensitivity and specificity of 89% and 69% respectively. The approach used here is sensitive and specific at matching these drops (∼94% and ∼95% respectively), which will provide a clinical foundation for use of the algorithm in the clinic. The precise features of the complex ECochG signal that provide the highest accuracy in detecting trauma are under evolving debate, with some improvements shown when including features such as CM latency [35] and ANN:CM ratio [36]. In the model developed here, including these features resulted in only a small improvement in detection rate (<10%).

In earlier studies optimising trauma detection, for example, in [36], typically an observational approach was used, comparing the peak CM response immediately prior to a drop to that at the nadir of the drop. However, at this point, it may be too late to initiate intervention. The goal of the present study was to facilitate the provision of rapid, intra-operative feedback as and when a drop occurs. The speed of detection of the proposed method is suitable for this purpose (∼0.75ms on a computer with a 2.11 GHz processor). Rapid real-time feedback not only leads to quicker intervention but also assists in consistent and smoother insertion, which has been proven to reduce trauma [37].

While this study improved the accuracy of CM drop detection over a basic assessment of CM amplitude, it still relied on human assessment as the ground truth. It remains to be seen if methods based on classification such as that explored here, or other machine learning methods (such as change detection and recurrent neural networks), could be utilsed to surpass the human observer in detecting traumatic events.

Although the use of cross-validation ensured the robustness of the models, they were trained/tested on a relatively small dataset. To ensure their applicability on a wide range of patients, they need to be retrained on larger datasets as and when they become available. Also, limits of performance where the models may fail need to be explored and safeguards implemented to handle such situations, prior to practical use in the operating theatre.

## Conclusion

In this paper, we introduced a framework for detecting trauma during cochlear implant surgery using ECochG data. The process thus discussed consisted of 3 steps: feature detection, classification, and post-processing. All algorithms were specifically designed to enable real-time trauma detection. We achieved high performance results: ∼94% and ∼95% sensitivity and specificity respectively. The average prediction time was less than ∼0.75ms, indicating its viability to be used during surgery. We anticipate that the inclusion of automatic trauma detection in ECochG will improve its utility and scalability.

## References

[1] Vos T, Abajobir AA, Abate KH, Abbafati C, Abbas KM, Abd-Allah F, et al. Global, regional, and national incidence, prevalence, and years lived with disability for 328 diseases and injuries for 195 countries, 1990–2016: a systematic analysis for the Global Burden of Disease Study 2016. The Lancet. 2017;390(10100):1211–1259. doi: 10.1016/S0140-6736(17)32154-2 PMC5605509 28919117

[2] Livingston G, Sommerlad A, Orgeta V, Costafreda SG, Huntley J, Ames D, et al. Dementia prevention, intervention, and care. The Lancet. 2017;390(10113):2673–2734. 28735855 10.1016/S0140-6736(17)31363-6

[3] Wilson BS, Tucci DL, Merson MH, O’Donoghue GM. Global hearing health care: new findings and perspectives. The Lancet. 2017;390(10111):2503–2515. doi: 10.1016/S0140-6736(17)31073-5 28705460

[4] Davis AC, Hoffman HJ. Hearing loss: rising prevalence and impact. Bulletin of the World Health Organization. 2019;97(10):646. 31656325 10.2471/BLT.19.224683 PMC6796666

[5] Emmett SD, Sudoko CK, Tucci DL, Gong W, Saunders JE, Advocacy GHHLE, et al. Expanding Access: Cost-effectiveness of Cochlear Implantation and Deaf Education in Asia. Otolaryngology–Head and Neck Surgery. 2019;161(4):672–682. doi: 10.1177/0194599819849917 31210566

[6] Sorkin DL. Cochlear implantation in the world’s largest medical device market: utilization and awareness of cochlear implants in the United States. Cochlear implants international. 2013;14(sup1):S12–S4. doi: 10.1179/1467010013Z.00000000076 23453146 PMC3663290

[7] Gifford RH, Dorman MF, Skarzynski H, Lorens A, Polak M, Driscoll CL, et al. Cochlear implantation with hearing preservation yields significant benefit for speech recognition in complex listening environments. Ear and hearing. 2013;34(4):413. doi: 10.1097/AUD.0b013e31827e8163 23446225 PMC3742689

[8] D’Haese PS, Van Rompaey V, De Bodt M, Van de Heyning P. The knowledge and beliefs regarding practical aspects of cochlear implants: a study of otorhinolaryngologists in a secondary setting in a multi-country study. Cochlear Implants International. 2018;19(1):14–21. doi: 10.1080/14670100.2017.1385141 28992743

[9] Dillon B, Pryce H. What makes someone choose cochlear implantation? An exploration of factors that inform patient decision making. International Journal of Audiology. 2020;59(1):24–32. doi: 10.1080/14992027.2019.1660917 31500481

[10] Radeloff A, Shehata-Dieler W, Scherzed A, Rak K, Harnisch W, Hagen R, et al. Intraoperative monitoring using cochlear microphonics in cochlear implant patients with residual hearing. Otology & Neurotology. 2012;33(3):348–354. doi: 10.1097/MAO.0b013e318248ea86 22377649

[11] Campbell L, Kaicer A, Sly D, Iseli C, Wei B, Briggs R, et al. Intraoperative real-time cochlear response telemetry predicts hearing preservation in cochlear implantation. Otology & Neurotology. 2016;37(4):332–338. doi: 10.1097/MAO.0000000000000972 26859542

[12] Dalbert A, Huber A, Veraguth D, Roosli C, Pfiffner F. Assessment of cochlear trauma during cochlear implantation using electrocochleography and cone beam computed tomography. Otology & Neurotology. 2016;37(5):446–453. doi: 10.1097/MAO.0000000000000998 26945317

[13] O’Leary S, Briggs R, Gerard JM, Iseli C, Wei BP, Tari S, et al. Intraoperative observational real-time electrocochleography as a predictor of hearing loss after cochlear implantation: 3 and 12 month outcomes. Otology & Neurotology. 2020;41(9):1222. doi: 10.1097/MAO.0000000000002773 32925842 PMC7497893

[14] Bester C, Collins A, Razmovski T, Weder S, Briggs RJ, Wei B, et al. Electrocochleography triggered intervention successfully preserves residual hearing during cochlear implantation: Results of a randomised clinical trial. Hearing research. 2021; p. 108353. doi: 10.1016/j.heares.2021.108353 34600798

[15] Dallos P, Cheatham MA, Ferraro J. Cochlear mechanics, nonlinearities, and cochlear potentials. The Journal of the Acoustical Society of America. 1974;55(3):597–605. doi: 10.1121/1.1914570 4819860

[16] Ruben R, Bordley J, Lieberman A. Cochlear potentials in man. The Laryngoscope. 1961;71(10):1141–1164. doi: 10.1288/00005537-196110000-00001 14494859

[17] Patuzzi R, Yates G, Johnstone B. Outer hair cell receptor current and sensorineural hearing loss. Hearing research. 1989;42(1):47–72. doi: 10.1016/0378-5955(89)90117-2 2684949

[18] Ruben R, Sekula J. Electrical potentials of the organ of hearing. Otolaryngologia polska The Polish otolaryngology. 1961;15:401–406. 14494862

[19] Palmer A, Russell I. Phase-locking in the cochlear nerve of the guinea-pig and its relation to the receptor potential of inner hair-cells. Hearing research. 1986;24(1):1–15. doi: 10.1016/0378-5955(86)90002-X 3759671

[20] Weinberger N, Kitzes L, Goodman D. Some characteristics of the ‘auditory neurophonic’. Experientia. 1970;26(1):46–48. doi: 10.1007/BF01900383 5412293

[21] Laszlo C, Gannon R, Milsum J. Measurement of the Cochlear Potentials of the Guinea Pig at Constant Sound-Pressure Level at the Eardrum. I. Cochlear-Microphonic Amplitude and Phase. The Journal of the Acoustical Society of America. 1970;47(4B):1063–1070. doi: 10.1121/1.1912006 5443153

[22] Santarelli R, Scimemi P, Dal Monte E, Arslan E. Cochlear microphonic potential recorded by transtympanic electrocochleography in normally-hearing and hearing-impaired ears. Acta otorhinolaryngologica italica. 2006;26(2):78. 16886850 PMC2639978

[23] Breiman L. Random forests. Machine learning. 2001;45(1):5–32. doi: 10.1023/A:1010933404324

[24] Fisher RA. The use of multiple measurements in taxonomic problems. Annals of eugenics. 1936;7(2):179–188. doi: 10.1111/j.1469-1809.1936.tb02137.x

[25] Piryonesi SM, El-Diraby TE. Role of Data Analytics in Infrastructure Asset Management: Overcoming Data Size and Quality Problems. Journal of Transportation Engineering, Part B: Pavements. 2020;146(2):04020022.

[26] Cristianini N, Shawe-Taylor J, et al. An introduction to support vector machines and other kernel-based learning methods. Cambridge university press; 2000.

[27] Dasarathy B. NN concepts and techniques; 1991.

[28] Hopfield JJ. Neural networks and physical systems with emergent collective computational abilities. Proceedings of the national academy of sciences. 1982;79(8):2554–2558. doi: 10.1073/pnas.79.8.2554 6953413 PMC346238

[29] Freund Y, Schapire RE. A decision-theoretic generalization of on-line learning and an application to boosting. Journal of computer and system sciences. 1997;55(1):119–139. doi: 10.1006/jcss.1997.1504

[30] Seiffert C, Khoshgoftaar TM, Van Hulse J, Napolitano A. RUSBoost: Improving classification performance when training data is skewed. In: 2008 19th International Conference on Pattern Recognition. IEEE; 2008. p. 1–4.

[31] Breiman L. Bagging predictors. Machine learning. 1996;24(2):123–140. doi: 10.1007/BF00058655

[32] Altman DG, Bland JM. Diagnostic tests. 1: Sensitivity and specificity. BMJ: British Medical Journal. 1994;308(6943):1552. doi: 10.1136/bmj.308.6943.1552 8019315 PMC2540489

[33] Ramos-Macias A, O’Leary S, Ramos-deMiguel A, Bester C, Falcon-González JC. Intraoperative intracochlear electrocochleography and residual hearing preservation outcomes when using two types of slim electrode arrays in cochlear implantation. Otology & Neurotology. 2019;40(5S):S29–S37. doi: 10.1097/MAO.0000000000002212 31225820

[34] Weder S, Bester C, Collins A, Shaul C, Briggs RJ, O’Leary S. Toward a Better Understanding of Electrocochleography: Analysis of Real-Time Recordings. Ear and Hearing. 2020;. doi: 10.1097/AUD.0000000000000871 33136631

[35] Sijgers L, Pfiffner F, Grosse J, Dillier N, Koka K, Röösli C, et al. Simultaneous Intra-and Extracochlear Electrocochleography During Cochlear Implantation to Enhance Response Interpretation. Trends in Hearing. 2021;25:2331216521990594. doi: 10.1177/2331216521990594 33710919 PMC7958165

[36] Giardina CK, Brown KD, Adunka OF, Buchman CA, Hutson KA, Pillsbury HC, et al. Intracochlear electrocochleography: response patterns during cochlear implantation and hearing preservation. Ear and hearing. 2019;40(4):833–848. doi: 10.1097/AUD.0000000000000659 30335669 PMC6534483

[37] Rajan GP, Kontorinis G, Kuthubutheen J. The effects of insertion speed on inner ear function during cochlear implantation: a comparison study. Audiology and Neurotology. 2013;18(1):17–22. doi: 10.1159/000342821 23006502

